# Sea surface temperature predicts the movements of an Arctic cetacean: the bowhead whale

**DOI:** 10.1038/s41598-018-27966-1

**Published:** 2018-06-25

**Authors:** Philippine Chambault, Christoffer Moesgaard Albertsen, Toby A. Patterson, Rikke G. Hansen, Outi Tervo, Kristin L. Laidre, Mads Peter Heide-Jørgensen

**Affiliations:** 1 Greenland Institute of Natural Resources, Strandgade 91, 2, DK-1401 Copenhagen, Denmark; 2 National Institute of Aquatic Resources, Technical University of Denmark, DK-2800 Kongens Lyngby, Denmark; 3 CSIRO Oceans and Atmosphere, GPO Box 1538, Hobart, TAS-7000 Australia; 4 Polar Science Center, Applied Physics Laboratory, University of Washington, 1013 NE, 40th Street, Seattle, WA-98105-6698 United States of America

## Abstract

The effects of climate change constitute a major concern in Arctic waters due to the rapid decline of sea ice, which may strongly alter the movements and habitat availability of Arctic marine mammals. We tracked 98 bowhead whales by satellite over an 11-year period (2001–2011) in Baffin Bay - West Greenland to investigate the environmental drivers (specifically sea surface temperature and sea ice) involved in bowhead whale’s movements. Movement patterns differed according to season, with aggregations of whales found at higher latitudes during spring and summer likely in response to sea-ice retreat and increasing sea temperature (SST) facilitated by the warm West Greenland Current. In contrast, the whales moved further south in response to sea temperature decrease during autumn and winter. Statistical models indicated that the whales targeted a narrow range of SSTs from −0.5 to 2 °C. Sea surface temperatures are predicted to undergo a marked increase in the Arctic, which could expose bowhead whales to both thermal stress and altered stratification and vertical transport of water masses. With such profound changes, bowhead whales may face extensive habitat loss. Our results highlight the need for closer investigation and monitoring in order to predict the extent of future distribution changes.

## Introduction

In response to climate change, modifications of the environmental conditions encountered by marine species might strongly alter their movements and consequently their habitat^1–3^. This is especially true for species inhabiting Arctic regions, where the effects of climate change are of major concern due to the decline of sea ice, i.e. a decrease of 3 to 4% per decade highlighted in the past 29 years^4,5^. Among the marine species that year-round inhabit Arctic waters, 11 are marine mammals (i.e. six pinnipeds, the walrus, three cetaceans and the polar bear)^6–8^. Sea ice provides access to air and platforms for haul-out, making some of these Arctic marine mammals strongly dependent on sea ice to support their life-history events^6–8^. For example, ribbon, spotted, harp and hooded seals breed on pack ice^9–11^, whereas ringed and bearded seals use stable ice for raising their pups and molting^7,12,13^.

Associations between sea ice and their movements have also been highlighted for Artic cetaceans. The narwhal population of Davis Strait and Baffin Bay performs seasonal movements that are directly related to the retreat and advance of sea ice^14^. Heide-Jørgensen *et al*.^15^ have shown that sea-ice loss has an effect on beluga whales’ distribution in West Greenland, as with sea-ice retreat, beluga whales abandon the coastal ice-free areas and follow the retreating sea-ice to the west. Additionally, several studies conducted in the Bering Sea have concluded that bowhead whales are associated with areas of high sea-ice concentration (e.g. up to >90%^16–19^). However, unlike seals, polar bear and walrus, Arctic cetaceans (narwhals, belugas and bowhead whales) are not “sea-ice obligates”. They have evolved to deal with high concentrations of sea ice and they do not necessarily depend on sea ice, and in some cases, sea ice acts more like a barrier to potential feeding grounds. By structuring the ecosystem and therefore influencing prey availability^7^, the effect of sea ice on the movements of Arctic cetaceans is probably indirect, and the distribution of these species might be more influenced by thermal conditions and their cascading effects on the food chain rather than on the sea ice *per se*. In this study we address this question directly and attempt to determine which of a suite of habitat predictors best predict bowhead whale distributions.

Although the main factor influencing the geographical range of many lower latitude cetaceans is water temperature^20–22^, the relationships between Arctic cetaceans and this variable have been poorly investigated, with only a few studies focusing on beluga and bowhead whales. From May to July, most beluga whales move into lagoons and estuaries in warmer waters to feed, bear their calves and moult^23–25^. Beyond the summer season, Bailleul *et al*.^26^ have demonstrated that the timing of migration in this species was related to sea surface temperatures (SSTs) conditions, with a later summer departure during summers with warmers SSTs. Regarding bowhead whales, a recent study conducted on the Pacific stock has demonstrated an avoidance of warmer waters coming from the Alaskan Coastal current^27^.

Among the five stocks of bowhead whales^28^, the population of Baffin Bay-Davis Strait occupies a wide geographical range spreading from the Canadian High Arctic to West Greenland^29–31^. Several studies involving satellite tracking of individuals from this population have shown migratory patterns for this species, and noted that an aggregation occurs in Disko Bay, West Greenland, from February to June^15,29,32^. In this area, the strong inter-annual site fidelity of bowhead whales can be partly explained by high concentrations of copepods, making this site an important foraging ground^33–35^. However, the local biomass of their preferred prey peaks in June^36^, indicating that the whales leave the foraging ground of Disko Bay prior to the peak availability of copepods. The reason for this mismatch is unclear, and it has been proposed that ocean temperature may be involved, either by being outside the range commonly used by this species or by forcing water column stratification in spring, which subsequently changes the density of prey^37^. Given that the West Greenland Current brings warm North Atlantic waters to Disko Bay in spring^38–40^, and that the water masses on the West Greenland Shelf increase in temperature from April^41^, we expect that bowhead whales adapted to cold temperatures will avoid the increase spring temperature in Disko Bay.

To test this hypothesis, we used tracking data from 98 bowhead whales originating from the same population of Baffin Bay-Davis Strait over an 11 year-time-series (2001–2011), to investigate the role played by either the SST and/or sea ice in driving their seasonal movements. Track lines or dive data from some of the whales have been presented in previous publications (see Supplementary Material Table S1), but this is the first analysis of the habitat use based on the complete set of tracking data by combining the three tagging locations for this population. This large sample size and long time series will provide a reliable picture of the environmental variables involved in the habitat selection of this Arctic marine cetacean.

## Results

### Global distribution

The 98 bowhead whales from the Baffin Bay-Davis Strait population were tracked from three different locations. We obtained 826 ± 821 (mean ± SD) locations per whale (range: 2–4129), for a tracking duration ranging from 1 day (#6335_11) to 489 days (#27262_10, mean ± SD: 110 ± 97 days) – see Supplementary Information Table S1. The total distance travelled varied from 5 km (#7617_11) to 15,230 km (#37227_10, mean ± SD: 3,118 ± 2,815 km). The actual speed ranged from 0.7 to 3.7 km.h^−1^ (#7925_09 vs. #27258_09, respectively, mean ± SD: 1.5 ± 0.3 km.h^−1^).

The estimated length of the tagged whales ranged from 8.5 to 18 m, measuring on average (mean ± SD) 14.5 ± 2.0 m. The males were slightly but significantly longer (mean ± SD: 15.7 ± 1.7 m, n = 19) than the females (mean ± SD: 14.8 ± 1.9 m, n = 47, Kruskal-Wallis rank sum test, χ² = 8,081, *p* < 0,001). The estimated length of the whales differed significantly according to the tagging location, being longer for those tagged in Disko Bay (mean ± SD: 14.8 ± 1.7 m, n = 83), than those tagged in Foxe Basin (mean ± SD: 13.8 ± 2.6 m, n = 11), or in Cumberland Sound (mean ± SD: 10.2 ± 0.8 m, n = 4, Kruskal-Wallis rank sum test, χ² = 3,238, *p* < 0,001).

**Figure 1:**
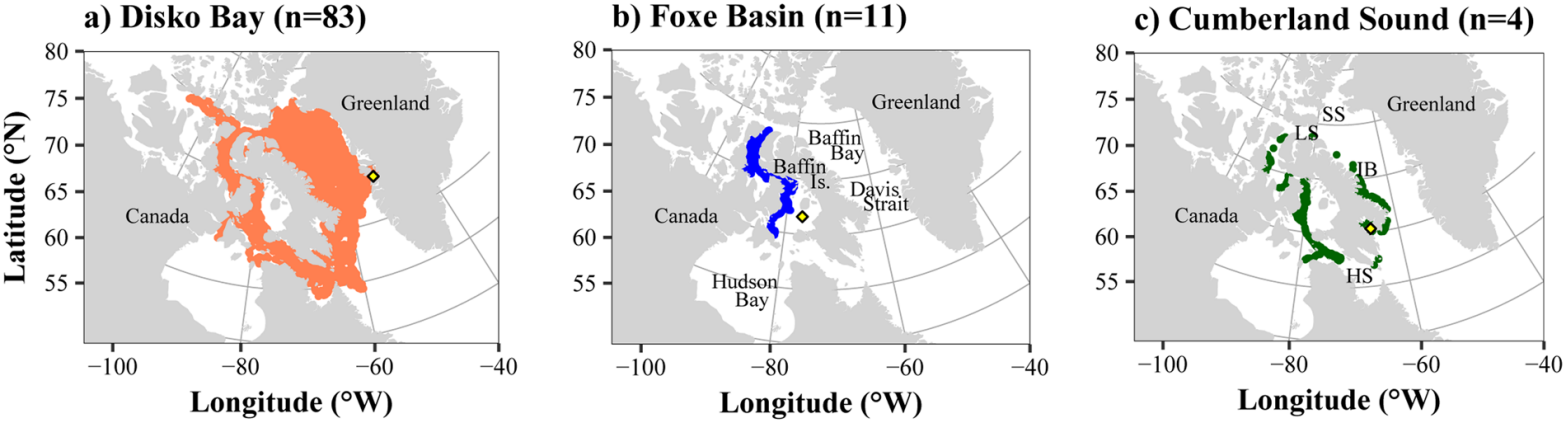
Locations of the 98 bowhead whales tracked from Disko Bay (**a**), Foxe Basin (**b**) and Cumberland Sound (**c**). The sample size (number of tracked individuals) is noted in parentheses of each panel and the tagging site with a yellow diamond. Baffin Is. refers to Baffin Island, SS to Smith Sound, LS to Lancaster Sound, IB to Isabella Bay and HS to Hudson Strait. Maps were generated using R software version 3.4.3. (R Core Team (2017). R: A language and environment for statistical computing. R Foundation for Statistical Computing, Vienna, Austria. http://www.R-project.org/).

The whales tracked from Disko Bay (n = 83) dispersed largely across Baffin Bay, all around Baffin Island and within the fjords of the Canadian Arctic Archipelago (CAA, Fig. 1a). Only few whales from Disko Bay entered the North Water in Smith Sound. In contrast, the individuals tracked from Foxe Basin (n = 11) remained within the CAA, north-west of Baffin Island (Fig. 1b), and those tracked from Cumberland Sound occupied the west and south-eastern parts of Baffin Island (Fig. 1c).

### Seasonal patterns and high-use areas

**Figure 2:**
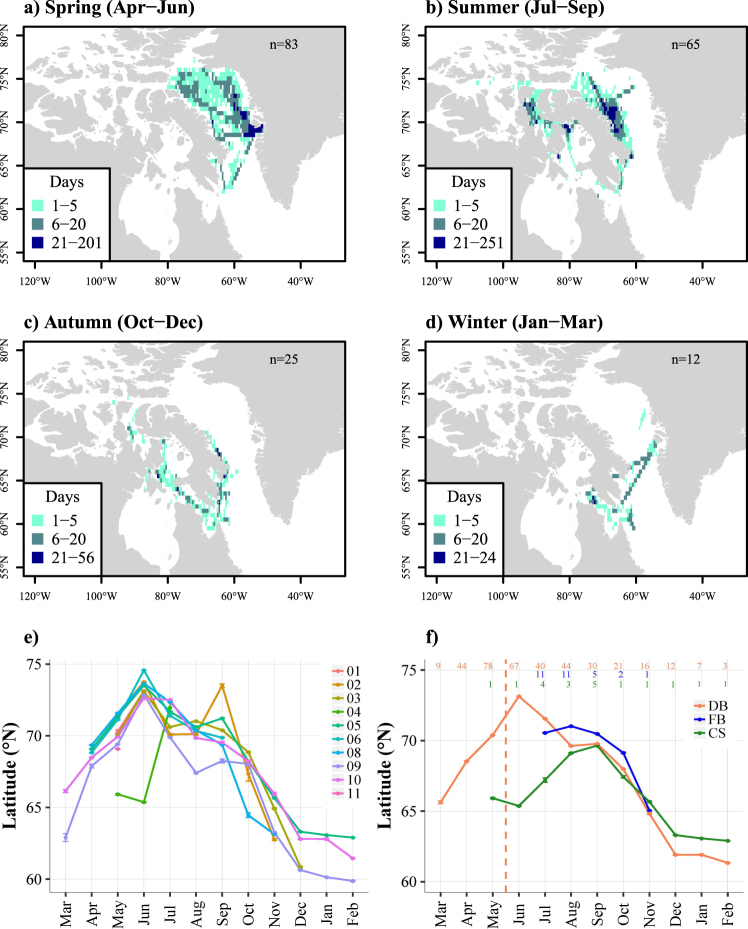
Bowhead whale habitat use maps in spring (**a**), summer (**b**), autumn (**c**) and winter (**d**), and monthly means (±SE) of latitude over months for each tracking year (**e**), and for each tagging site (**f**): Disko Bay (DB), Foxe Basin (FB) and Cumberland Sound (CS). Colour scale is indicative of the number of cumulative days the whales had pass through each grid cell of 0.5 × 0.5 decimal degree. The number of tracked individuals per season is noted at the top right of each panel. The numbers at the top of Fig. 3b refer to the sample size for each month and each tagging location, i.e. the number of the whales tracked, and the dotted line in Fig. 3f stands for the mean departure date from Disko Bay. Maps were generated using R software version 3.4.3. (R Core Team (2017). R: A language and environment for statistical computing. R Foundation for Statistical Computing, Vienna, Austria. http://www.R-project.org/).

When pooling the three tagging locations together, a general seasonal pattern of movement was observed. In spring (from April to June, n = 83), the whales remained within Baffin Bay, aggregating close to Disko Bay (Fig. 2a). In summer (from July to September, n = 65), the individuals remained on the north-eastern and north-western parts of Baffin Island, close to Isabella Bay and the CAA, respectively (Fig. 2b). In autumn (from October to December, n = 25), the whales occupied the waters south and southwest of Baffin Island (Fig. 2c), whereas in winter (from January to March, n = 12), the 12 tracked whales remained south and southeast of Baffin Island (Fig. 2d).

Seasonal patterns in latitudinal movements were observed across both years and according to tagging location (Fig. 2e,f). Globally, from March to June, the mean latitude used by the whales increased to a maximum in June (mean: 72.2°N), and then decreased to a minimum in February (mean: 61.4°N) – see Fig. 2e. This pattern was observed irrespective of tagging location or decreasing sample sizes of individuals tracked over time (Fig. 2f). The whales from Foxe Basin used the area between a maximum latitude in August (mean: 71.0°N) and a minimum latitude in November (mean: 65.0°N), and whales from Cumberland Sound travelled between (mean) 62.8°N in February and (mean) 69.6°N in September.

### Movements in relation to SST

**Figure 3:**
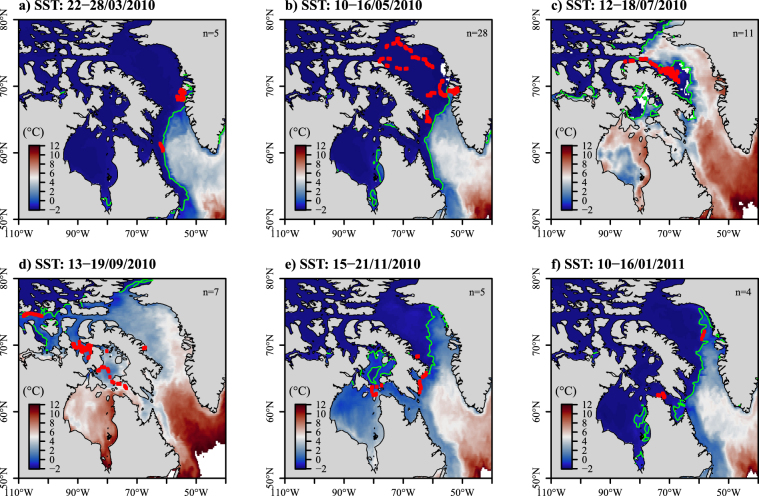
Maps of the weekly averaged SST (in °C) derived from Copernicus in March (**a**), May (**b**), July (**c**), September (**d**), November (**e**) and January (**f**) 2010–2011. Positions of bowhead whales are indicated with red dots and the green lines stand for the −1 °C isotherm (freezing point). The number of tracked individuals per week is noted at the top right of each panel. Maps were generated using R software version 3.4.3. (R Core Team (2017). R: A language and environment for statistical computing. R Foundation for Statistical Computing, Vienna, Austria. http://www.R-project.org/).

Associations between the whales’ movements and the SST were observed over the 10 tracking years. As warmer surface waters moved from the south-western part of Greenland in March, whales departed from Disko Bay and headed west across Baffin Bay (Fig. 3a,b). In July, the whales remained in the north-western part of Baffin Bay (Fig. 3c), north of the −1 °C isotherm, where the SST remained colder (mean: −0.3 °C) than in the middle of Baffin Bay (>3 °C) – see Fig. 3c.

**Figure 4:**
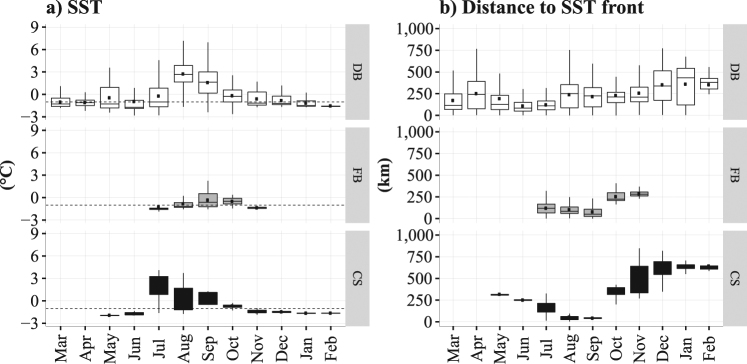
Box plots of the SST (**a**, in °C) and distance to the closest SST front (**b**, in km) over months, extracted at each whale’s location originating from each tagging site: Disko Bay (DB), Foxe Basin (FB) and Cumberland Sound (CS). The black dots in each box plot refer to the mean value for each month. The dotted line in (**a**) refers to the −1 °C isotherm (i.e. freezing point).

With the autumn advance of sea ice and the decreased SST, the whales moved further south along the east coast of Baffin Island (Fig. 3d,e). Overall throughout the year, these whales experienced small variations in terms of SST (range: −1.5 to 8.7 °C), with a minimum average observed in February (mean: −1.5 °C) and a maximum average in August (mean: 1.7 °C) – see Fig. 4a. The whales tracked from Disko Bay remained mostly close to the −1 °C isotherm, except between August (mean: 2.6 °C) and September (mean: 1.6 °C) – see Fig. 4a top. Those tracked from Foxe Basin experienced colder and more stable temperatures (Fig. 4a middle), whereas those tracked from Cumberland Sound remained globally below the −1 °C isotherm, except between July (mean: 1.6 °C) and September (mean: 0.3 °C) – see Fig. 4a bottom.

**Figure 5:**
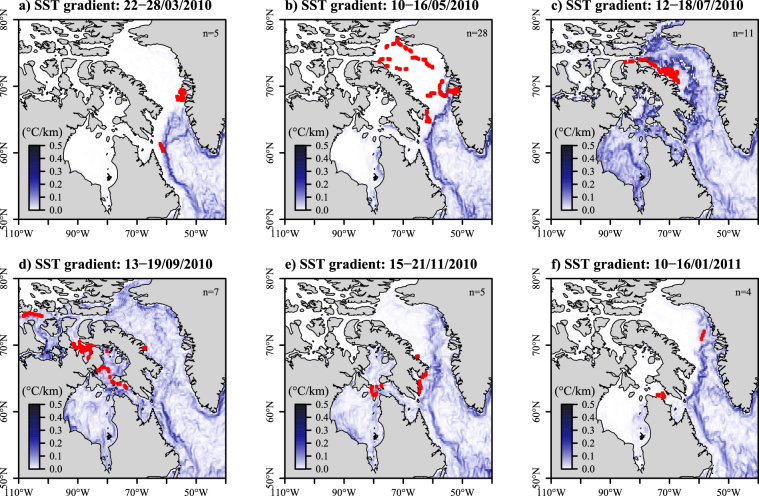
Maps of the weekly averaged SST gradient (in °C/km) derived from Copernicus SST in March (**a**), May (**b**), July (**c**), September (**d**), November (**e**) and January (**f**) 2010–2011. Positions of bowhead whales are indicated with red dots. The number of tracked individuals per week is noted at the top right of each panel. Maps were generated using R software version 3.4.3. (R Core Team (2017). R: A language and environment for statistical computing. R Foundation for Statistical Computing, Vienna, Austria. http://www.R-project.org/).

The relationships between the whales’ movements and the SST front are shown in Figs 4 and 5. As the SST gradient moved north-westward in Baffin Bay, the distance between the whale’s locations and the closest SST front was low between May to July (mean: 139 km) for the whales tagged in Disko Bay (Figs 4b top, 5b and 5c). The individuals tagged at the two other sites did not exhibit any particular pattern with the SST front (Fig. 4b middle and bottom). The SST front then progressively disappeared from August to October (Fig. 5d).

### Movements in relation to sea ice

**Figure 6:**
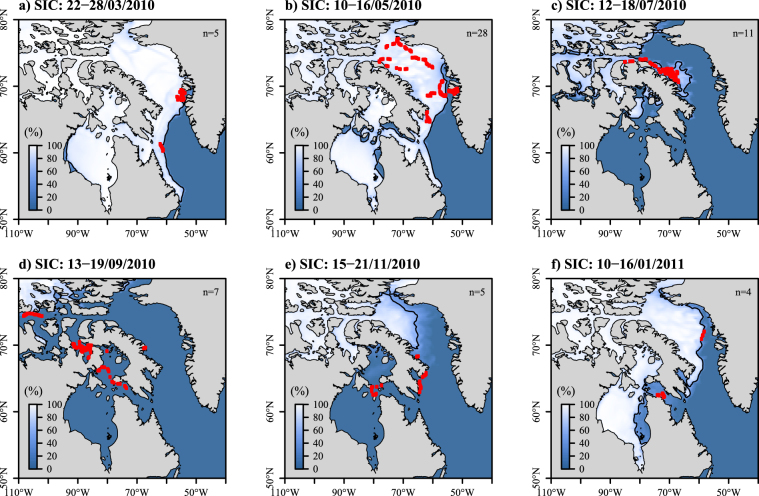
Maps of the weekly averaged sea ice concentration (SIC, in %) derived from Copernicus in March (**a**), May (**b**), July (**c**), September (**d**), November (**e**) and January (**f**) 2010–2011. Positions of bowhead whales are indicated with red dots and the black solid lines stands for the 50% SIC contour (i.e. sea-ice edge isoline). The number of tracked individuals per week is noted at the top right of each panel. Maps were generated using R software version 3.4.3. (R Core Team (2017). R: A language and environment for statistical computing. R Foundation for Statistical Computing, Vienna, Austria. http://www.R-project.org/).

**Figure 7:**
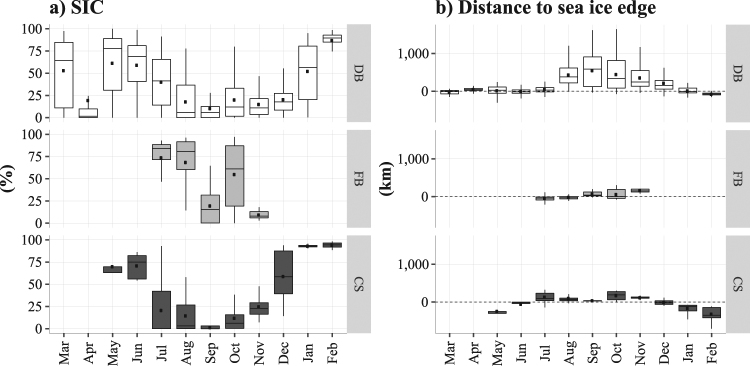
Box plots of the SIC (**a**, in %) and the distance to sea-ice edge (**b**, in km) over months, extracted at each whale’s locations originating from each tagging site: Disko Bay (DB), Foxe Basin (FB) and Cumberland Sound (CS). The black dots in each box plot refer to the mean value for each month. Negative values in (**b**) correspond to locations associated with SIC ≥50%, and positive values to locations in the open water (SIC < 50%). The dotted line in (**b**) refers to the sea-ice edge.

March is the coldest month where annual sea-ice concentrations (SIC) in Baffin Bat (including Disko Bay) reaches values between 62 and 100% (Fig. 6a). From May to June, the whales left Disko Bay as sea ice started melting (Fig. 6b). Indeed, SIC globally decreased from May (58%) to September (mean: 11%) for the whales of Disko Bay (Fig. 7a top).

During summer (July to September), the whales used waters associated with lower SIC in Disko Bay and Cumberland Sound (Fig. 7a top and bottom), however not in Foxe Basin, where SIC was still high in August (mean: 69%) - see Fig. 7a middle. With autumn advance from October, SIC increased again within the study area (Fig. 7a), pushing the whales to migrate further south of Baffin Island (Fig. 6e).

The whales from Disko Bay tended to follow the sea-ice edge (defined as 50% isoline of SIC) during spring and winter (Fig. 7b top). In contrast, during summer and autumn, when sea ice concentration was low at the locations selected by the whales, thes whales were mainly found in open ice-free waters (Fig. 6d,e). Unlike the whales from Disko Bay, the whales originating from Foxe Basin remained close to sea-ice edge throughout the tracking period (Fig. 7b middle). During spring and winter, the whales tagged in Cumberland Sound were associated with negative values of distance to sea-ice edge, i.e. they spent this period within areas surrounded by sea ice (Fig. 7b bottom).

### Habitat suitability model

**Figure 8:**
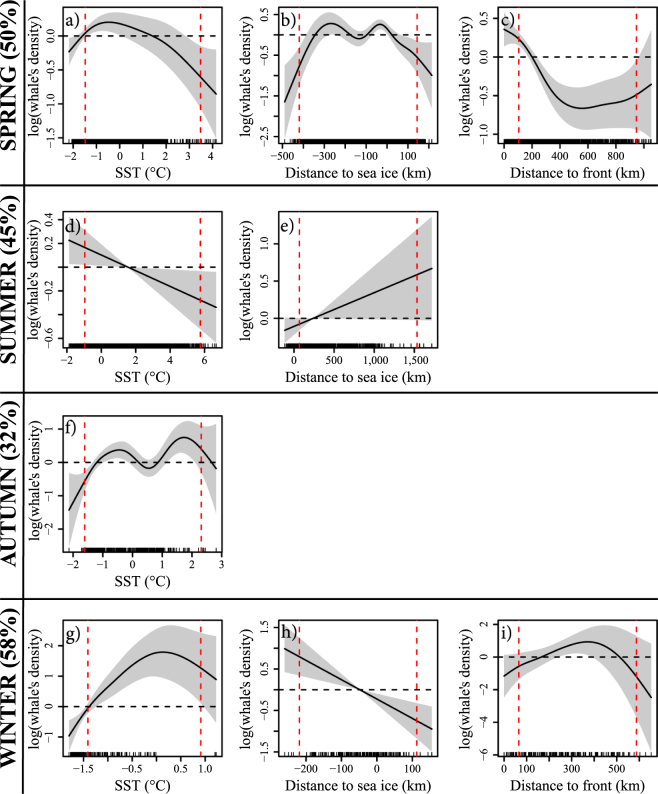
Relationships between bowhead whale’s density (y axis) and their associated variables (e.g. SST, SIC, distance to sea ice and distance to SST front) obtained from the four seasonal GAMs: spring (a,b,c), summer (d,e), autumn (f) and winter (g,h,i). The best model and its associated variables are presented for each season with its explained deviance in parentheses. The solid black line in each plot is the smooth function estimate and the shaded regions refer to the approximate 95% confidence intervals. The y axis represents the response variable expressed in log scale. Positive values on the y axis indicate a high probably of whale’s presence, and conversely. The horizontal dotted lines indicate no effect of the environmental variable. The red vertical lines refer to the 10^th^ and 90^th^ quantiles of the tracking dataset, i.e. best models fit between these two lines.

For the habitat suitability, explained deviances ranged between 32% in autumn to 58% in winter (Fig. 8). Due to collinearity, some variable combinations were excluded and all combinations are presented in Supplementary Information Table S2. For the four seasonal models, the common variable associated with the best model (lower AIC, higher explained deviance and the most parsimonious) was SST. The best model for spring and winter contained three covariates: SST, distance to sea-ice edge and distance to SST front.

Distance to SST front was a significant covariate for spring and winter. In spring, the whale’s density (i.e. mean number of whales per cell grid) decreased with the distance to SST front (Fig. 8c), meaning that the whales were mostly found close to the front. In contrast, in winter there was a positive relationship between the whale’s density and SST, indicating that bowhead whales were mostly found far from the front (Fig. 8i).

Distance to sea-ice edge was a significant explanatory variable in spring, summer and winter. There was a positive relationship between the distance to sea-ice edge and the whale’s density in summer (Fig. 8e), with the highest densities in open waters (i.e. positive values of distance to sea-ice edge). Conversely, the whale’s densities were higher for low values of distance to sea-ice edge both in spring and winter (Fig. 8b,h), suggesting higher whale’s densities within the sea ice (i.e. negative values of distance to sea-ice edge).

The SST variable was retained in the four seasonal models. Except in summer, when there was a negative relationship between SST and bowhead whale’s density (Fig. 8d), for the three other seasons there was an almost “dome shaped” relationship with SST, indicating a negative response of the whale’s density at very cold and very warm temperatures (Fig. 8a,f,g). Furthermore, temperature optima ranged between −0.5 and 2 °C (in spring and autumn, respectively), showing a very narrow temperature range for this species.

## Discussion

The use of a large tracking dataset (n = 98) together with environmental covariates provided novel information on the crucial role played by the SST in driving the movements of a cetacean that resides year-round in Arctic waters. These results have also important implications for predicting the likely extent and distribution of habitat due to climate change.

The satellite tracking of the 98 bowhead whales showed different spatial patterns based on where they were tagged. The whales tagged in Disko Bay dispersed much more widely and used a broader geographical range than the whales tagged in Foxe Basin or Cumberland Sound. The whales tagged in Disko Bay were mainly adult females (range: 12–18 m), some of which might have been pregnant, and may have given birth during the spring migration^6,42^. This is in accordance with a previous study conducted in this area showing that the population of Disko Bay is dominated by females (78%) larger than 14 m^32^. On contrast, the whales from Foxe Basin were composed of large individuals (15–18 m) associated with smaller, and likely immature whales (10–11 m). Heide-Jørgensen *et al*.^32^ found similar results using skin samples, and several studies suggested that Foxe Basin may be an important nursing ground in late summer and early autumn for this species^43,44^. Finally, the whales originating from Cumberland Sound were composed of smaller individuals (8–11 m), presumably immature whales. Given that sexual maturity is reached at 12–14 m in length in this species^45^, we assume that immature whales might not be ready to travel to Disko Bay to utilize the relatively deep waters in the bay compared to more shallow areas in Foxe Basin and Cumberland Sound. Despite the unequal sample size between the three tagging locations, the present tracking dataset confirmed that bowhead whales segregate by sex, age and reproductive status. For the first time, this large tracking dataset was analysed by combining the three different tagging locations in Baffin Bay, providing a reliable picture of the bowhead whale’s movements in this region as well as highlighting pronounced seasonal patterns.

The seasonal pattern revealed by the whales’ tracks was characterized by north-south movements independent of tagging location. Despite the decreasing sample size through months (higher in spring and summer), all individuals reveal the same migration pattern, moving further north during the warm season (spring and summer), and moving south to lower latitudes during autumn and winter. This pattern is reinforced irrespective of tagging location, and is not an artefact of tag loss. In agreement with the study of Ferguson *et al*.^44^, we have shown that the whales tagged in Foxe Basin were associated with lower ice concentrations during autumn-winter (mean SIC: 44%) compared to summer (mean SIC: 62%). The selection of areas characterized by less ice concentration, thinner ice and smaller floes in winter might reduce the risk of ice entrapment and provide protection from killer whales^44^, especially for immature individuals that are dominant in Foxe Basin. A similar trend was observed for the whales tagged in Disko Bay which experienced lower concentrations of sea ice during autumn (mean SIC: 18%) than during summer (35%). Despite their ability to break thick ice (up to 60 cm thick^6,46^), bowhead whales in Baffin Bay generally followed the sea-ice edge, especially those departing from Disko Bay to reach Baffin Island. Although the marginal ice zone is known to be one of the most productive areas in the Arctic Ocean^47^, the bowhead whales were more associated with particular values of SST rather than the sea-ice edge, suggesting that the sea-ice edge is unlikely to be used as a foraging area by this species. Many studies conducted in Disko Bay reinforce this assumption as they showed a high biomass of copepods *Calanus* during spring^33,36,48,49^, which is the main prey of adult bowhead whales.

The migratory behaviour of bowhead whales has already been evidenced by previous studies on this population but for fewer individuals (n < 10 from Disko Bay^29,31^, and n = 27 from Foxe Basin^44^), and so far no study has tried to relate such a behaviour to sea surface temperature. The departure of bowhead whales from Disko Bay coincides with the inflow of warm waters coming from the Atlantic and transferred by the West Greenland Current^40^. Despite the sea-ice retreat removing the physical barrier for whales crossing Baffin Bay, it may be that an affinity for colder waters could explain the whales’ departure as SST increases. This hypothesis is supported by our findings from the habitat models that show a consistent relationship between SST and bowhead whale densities for all seasons, highlighting an optimum temperature varying from −0.5 °C in spring to 2 °C in autumn. A similar behaviour was observed for the bowhead whales from the Bering-Chukchi-Beaufort Sea population which apparently avoid the warm Alaskan Coastal Current^27^, although the authors suggest it was the lower densities of zooplankton in the warm waters that spurred the movements of the bowhead whales. In contrast, the bowhead whales in Baffin Bay abandon highly productive waters in Disko Bay just prior to the peak zooplankton bloom in the area^36^. This supports the observation that the whales’ departure from Disko Bay is not related to the availability of their prey but rather the preference to remain in cooler waters. Bowhead whales have extremely thick blubber layers, that in Disko Bay has been measured up to 36 cm^50^, providing insulation adequate to maintain steady thermal state in external temperatures as low as liquid oxygen^51^. This indicates that potential heat-stress or hyperthermia from swimming activity is likely a key factor influencing the bowhead whales’ movements away from warm water despite dense zooplankton concentrations. The whales capitalize on the zooplankton production in Disko Bay to increase the blubber layer as a protection against leaner periods, but the increased metabolism, with ensuing heat dissipation, required for long-distance movements drives the whales away from the area before the increase in water temperatures. The narrow range of surface temperature experienced by the whales (means: −1.6 to 2.7 °C) compare to the range observed in Disko Bay (means: −1.7 to 9 °C) supports this assumption. For bowhead whales with limited heat exchanging appendages (no dorsal fin) it seems unlikely that they can avoid hyperthermia in sea water of 9 °C (summer temperature in Disko Bay) without lowering metabolic rates to unrealistically low levels^51^.

Although the values of the explained deviances from the GAMs were comparable to what is commonly found in the literature when relating cetacean’s densities to environmental variables, e.g.^52–54^, the explained deviance of autumn was lower (32%). The diving and foraging behaviour of bowhead whales might explain such low percentages of explained deviance; whales follow the vertical migrations of copepods and can either feed on pelagic or hibernating zooplankton aggregations close to the sea floor^33,35^. Variability of the diving behaviour adds complexity to the environmental drivers and may limit the explanatory power of the additive models. Two studies have focused on the diving behaviour of bowhead whales in Baffin Bay^33,35^, but these were restricted to limited tracking durations (<30 d) and to Disko Bay. To better understand the three dimensional habitat use by this species during a complete migratory cycle (one year), additional diving data, together with *in situ* variables (temperature, salinity and fluorescence) to relate their diving behaviour to the properties of the water column are needed.

Given the complexity of the vertical processes occurring in the water column, the SST cannot alone explain the movements of bowhead whales and their departure from Disko Bay, but SST should however be considered as a proxy for what drives the movements of this arctic species in Baffin Bay. In essence, SST is used as a variable to capture both the thermal stress bowheads are exposed to, and the changes in stratification of the water masses. Simultaneous with the increase in SST, a distinct stratification of the otherwise well mixed water column takes place^55^. Copepods species like *C. hyperboreus* are hibernating at depths below those reached by the bowhead whales for most of the year where a pycnocline is also present^33^. The nutrient rich copepod *C. hyperboreus* has a very strict seasonal timing of vertical migrations and presence in the upper part of the water column accessible to bowheads^56^. In the Baffin Bay area, the other copepod species, *C. finmarchicus* is present throughout the winter at depths accessible to bowhead whales, but the larger and the more lipid rich *C. hyperboreus* is only available for a short period in late spring^57^. The main prey of bowhead whales – *C. hyperboreus* – hibernates at great depths and is only available to bowhead whales during spring when they make vertical migrations to the strictly seasonal phytoplankton bloom. It is therefore an important observation that bowhead whales in Baffin Bay abandon the Disko Bay feeding ground when sea temperature increases and before the peak abundance of *C. hyperboreus*.

Of the three Arctic cetaceans occurring in Baffin Bay, the bowhead whale undoubtedly has the greatest flexibility in movement patterns and habitat selection^58,59^. In the current study this is evidenced by the variable selection of summering grounds either east or west of Baffin Island, a flexibility that is not observed for the two other Arctic cetaceans (the narwhal and the beluga), that both have fairly predictable migratory patterns and high matrilineally driven site-fidelity^60,61^. Despite their relative plasticity in movement patterns, bowhead whales also function under strict environmental and physiological restraints. Evidently the sea ice should not be so thick that it prevents the whales from reaching the surface for breathing, sea ice appears to act more like a barrier restricting access to foraging areas rather than an asset for the whales. In some parts of their range in Baffin Bay, the bowhead whales will summer in areas that are completely devoid of sea ice (e.g. Isabella Bay) or have only scattered ice (15–50% coverage) as observed in most areas. What appears to be more important is that the sea surface temperature should be below 2 °C, which, in this region, often coincides with areas that have sea ice in winter and spring. The productive feeding area in Disko Bay^33,35^ is abandoned in late spring when the temperature rises and nothing suggests that prey availability is a driver for the departure from the bay. Our data are consistent with Disko Bay being used for spring feeding excursions for mainly mature females without calves while the core area used by the rest of the bowhead whale population (from Foxe Basin and Cumberland Sound) is around the Canadian Arctic Archipelago. However, feeding in Disko Bay by mature whales comes with the price that the area must be abandoned before sea temperatures rises and the whales, with replenished blubber deposits, will suffer from thermal stress. Long movements with high level of muscle activity will likely increase the risk of hyperthermia and travels across Baffin Bay may need to be initiated well in advance of the rising sea temperatures.

## Methods

### Study areas and tag deployment

Adult bowhead whales (>13 m in length^62^) were instrumented with satellite transmitters during March to May in 2001, 2002, 2003, 2005, 2006, and between 2008 and 2011 from Qeqertarsuaq (Disko Island, West Greenland, Fig. 1). Additional individuals were instrumented in July 2002 and 2003 in Foxe Basin (Canada), and in Cumberland Sound (Canada) from May to July 2004 to 2006. The transmitters were manufactured by Wildlife Computers (Redmond, Washington, USA) and modified for use on whales by MV Jensen (www.mikkelvillum.com). See Supplementary Information Table S1 for a list of tags and Heide-Jørgensen *et al*.^31^ and Nielsen *et al*.^63^ for a description of the tag configurations. The study was conducted under the general permission from the Government of Greenland to the Greenland Institute of Natural Resources for tagging baleen whales. The protocol for tagging of bowhead whales was reviewed and approved by the Danish Animal Welfare Committee (IACUC), Faculty of Health Sciences, University of Copenhagen.

Daily searches for whales were conducted in the northern part of Disko Bay, in Foxe Basin and in Cumberland Sound on days with good visibility and low sea state (Beaufort sea state 0 or 1) from small (6 m) boats with outboard engines (150 hp). As soon as a whale was spotted, the boats quickly moved close to the whale, and while the whale was diving, the boats spread out and waited for the whale to reappear. This procedure was repeated until it was possible to get close enough to apply the tag from the boats. Tagging was done either by using a custom-made 8 m long fiberglass pole or a pneumatic gun^64^. A skin biopsy for genetic studies and molecular sex determination was taken from each tagged whale either with the pole or with a crossbow, using genetic methods described in Heide-Jørgensen *et al*.^58^. Approximate length of the tagged whales was estimated by comparing the size of the whale with the length of the boats involved with the tagging. All tags started transmitting shortly after deployment when the conductivity switch was activated during submergence.

### Data pre-filtering

Data were relayed through the Argos Data Collection and Location System and decoded using Argos Message Decoder (DAP Ver. 3.0, build 114, Wildlife Computers). All statistical analyses were performed using R software version 3.4.3^65^. The filtering approach of Albertsen *et al*.^66^ was applied to the tracking data in order to improve the Argos locations. We then used the General Bathymetric Chart of the Oceans (GEBCO) database (http://www.gebco.net/, resolution 30 arc-second, ~1 km grid) to discard any remaining locations on land (2.8%). We also discarded the Argos locations associated with a speed of over 10 km.h^−1^ (0.05%), as well as “type Z” (i.e. invalid Argos-based) locations (0.01%).

### Environmental data

We extracted two environmental variables from both remote sensed data and model simulations to characterize the habitat of bowhead whales at their tracking locations. At each whale location, we extracted the daily associated Sea surface temperature (SST), sea ice concentration (hereafter SIC) from the *Global Ocean Physics Reanalysis Glorys S2V4* product (PHYS 001-025) at a resolution of 0.25° (from E.U. Copernicus Marine Service Information). Distance from sea-ice edge was calculated as the minimum distance between each whale location and the 50% sea ice concentration isoline (due to the ability of bowhead whales to break thick ice,^6,46^. Following Chambault *et al*.’s procedure^67^, we also generated maps of SST gradient (SSTgrad) derived from the SST over the whole study area. To identify the locations of oceanic fronts based on SST gradient, we used the areas with the highest SSTgrad magnitude (≥quantile 0.99) and calculated the distance between each whale position and the closest frontal zone identified.

### Identification of high-use areas

To assess seasonal patterns, one habitat use map per season was generated from tracking data by cumulating the number of days each individual had crossed each grid cell of 0.5 × 0.5 decimal degree. Seasons were defined as follow: spring from April to June, summer from July to September, autumn from October to December and winter from January to March. Indeed, the bowhead whales generally arrive in West Greenland between January and March, they spend April to mid-June in Disko Bay and abandon West Greenland after July. During July-September they roam widely in the CAA before moving towards their wintering ground Hudson Strait in October-December^34,68^.

### Habitat modelling

Given that the eastern part of Baffin Bay is more influenced by the warm waters coming from the North Atlantic than the western part^38–40^, we assumed that the whales wintering in Disko Bay would be more exposed to large changes in temperature and sea ice. For that reason, tracks from whales tagged in Foxe Basin and Cumberland Sound were not included in the habitat modelling analysis. To investigate whether SST or sea ice had an effect on bowhead whale movements, we constructed a series of Generalized Additive Models (GAMs) using the *mgcv* package in R^69,70^. The response variable was the number of bowhead whales that crossed each pixel of 0.5 × 0.5 decimal degree. Monthly grids of presence data (i.e. from the tracking data) were generated by aggregating the whale’s tracks for each month of each tracking year. The monthly averaged SST and SIC were extracted in each monthly grid cell, and the distance to the closest SST front and to the sea-ice edge were derived following the method described above. The response variable was over-dispersed counts. Accordingly, we employed GAMs with a Negative Binomial error distribution. Such a distribution can provide good fits when dealing with over-dispersed count data^71^. Four environmental predictors were used: SST, SIC, distance to sea-ice edge and distance to the closest SST front. For each season, the models with all possible combinations were compared using the Akaike Information Criterion (AIC) and their explained deviance, and including the variables with a Variance Inflation Factor below three to avoid collinearity^72^. Temporal autocorrelation was then tested for each selected model using the *acf* function in R. To account for spatial autocorrelation, spatial coordinates (i.e. longitude and latitude) were included in each model as an explanatory variable, and the tracking year was included as a factor to account for potential inter-annual effect.

## Electronic supplementary material

Summary of the horizontal movements of the 98 bowhead whales (Table S1) and AIC results showing all possible combinations for the seasonal GAMs (Table S2).

## References

[1] Parmesan C, Yohe G (2003). A globally coherent fingerprint of climate change impacts across natural systems. Nature.

[2] Thomas CD (2004). Extinction risk fromclimate change. Nature.

[3] Thomas, C. D. *et al*. Biodiversity conservation: Uncertainty in predictions of extinction risk/Effects of changes in climate and land use/Climate change and extinction risk (reply). *Nature* **430** (2004). 10.1038/nature02716 15237465

[4] Parkinson CL, Cavalieri DJ (2002). A 21 year record of Arctic sea-ice extents and their regional, seasonal and monthly variability and trends. Ann. Glaciol..

[5] Parkinson CL, Cavalieri DJ (2008). Arctic sea ice variability and trends, 1979–2006. J. Geophys. Res. Oceans.

[6] Perrin, W. F., Würsig, B. & Thewissen, J. G. M. *Encyclopedia of Marine Mammals*. (Academic Press, 2009).

[7] Kovacs KM, Lydersen C, Overland JE, Moore SE (2011). Impacts of changing sea-ice conditions on Arctic marine mammals. Mar. Biodivers..

[8] Laidre KL (2015). Arctic marine mammal population status, sea ice habitat loss, and conservation recommendations for the 21st century. Conserv. Biol..

[9] Kovacs, K. M. Hooded Seal: *Cystophora cristata*. In *Encyclopedia of Marine Mammals (Second Edition)* (eds Perrin, W. F., Würsig, B. & Thewissen, J. G. M.) 569–573, 10.1016/B978-0-12-373553-9.00132-2 (Academic Press, 2009).

[10] Lavigne, D. M. Harp Seal: *Pagophilus groenlandicus*. In *Encyclopedia of Marine Mammals (Second Edition)* (eds Perrin, W. F., Würsig, B. & Thewissen, J. G. M.) 542–546, 10.1016/B978-0-12-373553-9.00127-9 (Academic Press, 2009).

[11] Lowry, L. & Boveng, P. Ribbon Seal *Histriophoca fasciata*. In *Encyclopedia of Marine Mammals (Second Edition)* (eds Perrin, W. F., Würsig, B. & Thewissen, J. G. M.) 955–958, 10.1016/B978-0-12-373553-9.00218-2 (Academic Press, 2009).

[12] Hammill, M. O. Ringed Seal: *Pusa hispida*. In *Encyclopedia of Marine Mammals (Second Edition)* (eds Perrin, W. F., Würsig, B. & Thewissen, J. G. M.) 972–974, 10.1016/B978-0-12-373553-9.00221-2 (Academic Press, 2009).

[13] Kovacs, K. M. Bearded Seal: *Erignathus barbatus*. In *Encyclopedia of Marine Mammals (Second Edition)* (eds Perrin, W. F., Würsig, B. & Thewissen, J. G. M.) 97–101 (Academic Press, 2009).

[14] Laidre KL, Heide-Jørgensen MP (2005). Arctic sea ice trends and narwhal vulnerability. Biol. Conserv..

[15] Heide-Jørgensen MP (2010). *et al*. The effect of sea-ice loss on beluga whales (*Delphinapterus leucas*) in West Greenland. Polar Res..

[16] Moore, S. E. Distribution and movement. In *The bowhead whale* **2**, 313–386 (Allen Press Inc, 1993).

[17] Citta JJ (2014). Potential for bowhead whale entanglement in cod and crab pot gear in the Bering Sea. Mar. Mammal Sci..

[18] Citta J (2012). Winter Movements of Bowhead Whales (*Balaena mysticetus*) in the Bering Sea. Arctic.

[19] Citta JJ (2015). Ecological characteristics of core-use areas used by Bering–Chukchi–Beaufort (BCB) bowhead whales, 2006–2012. Prog. Oceanogr..

[20] Kaschner K, Watson R, Trites AW, Pauly D (2006). Mapping world-wide distributions of marine mammal species using a relative environmental suitability (RES) model. Mar. Ecol. Prog. Ser..

[21] MacLeod CD (2009). Global climate change, range changes and potential implications for the conservation of marine cetaceans: a review and synthesis. Endanger. Species Res..

[22] Fullard KJ (2000). Population structure of long-finned pilot whales in the North Atlantic: a correlation with sea surface temperature?. Mol. Ecol..

[23] Hansen, S. White whale (*Delphinapterus leucas*) distribution and abundance in relation to water temperature, salinity, turbidity and depth in the Churchill River estuary. (Laurentian University, 1988).

[24] Moore SE, Shelden KEW, Litzky LK, Mahoney BA, Rugh DJ (2000). Beluga, *Delphinapterus leucas*, Habitat Associations in Cook Inlet, Alaska. Mar. Fish. Rev..

[25] Smith AJ (2017). Beluga whale summer habitat associations in the Nelson River estuary, western Hudson Bay, Canada. PLOS ONE.

[26] Bailleul F, Lesage V, Power M, Doidge DW, Hammill MO (2012). Migration phenology of beluga whales in a changing Arctic. Clim. Res..

[27] Citta, J. J. *et al*. Oceanographic characteristics associated with autumn movements of bowhead whales in the Chukchi Sea. *Deep Sea Res. Part II Top. Stud. Oceanogr*, 10.1016/j.dsr2.2017.03.009 (2017).

[28] Rugh DJ (2003). A review of bowhead whale (*Balaena mysticetus*) stock identity. Cetacean Res. Manag..

[29] Heide-Jørgensen MP (2003). From Greenland to Canada in Ten Days: Tracks of Bowhead Whales, *Balaena mysticetus*, across Baffin Bay. Arctic.

[30] Heide-Jørgensen MP, Laidre KL, Quakenbush LT, Citta JJ (2012). The Northwest Passage opens for bowhead whales. Biol. Lett..

[31] Heide‐Jørgensen MP, Laidre KL, Jensen MV, Dueck L, Postma LD (2006). Dissolving stock discrteness with satellite tarcking: Bowhead whales in Baffin Bay. Mar. Mammal Sci..

[32] Heide-Jørgensen MP, Laidre K, Borchers D, Samarra F, Stern H (2007). Increasing abundance of bowhead whales in West Greenland. Biol. Lett..

[33] Laidre KL, Heide-Jørgensen MP, Nielsen TG (2007). Role of the bowhead whale as a predator in West Greenland. Mar. Ecol. Prog. Ser..

[34] Laidre KL, Heide-Jørgensen MP (2012). Spring partitioning of Disko Bay, West Greenland, by Arctic and Subarctic baleen whales. ICES J. Mar. Sci..

[35] Heide-Jørgensen MP, Laidre KL, Nielsen NH, Hansen RG, Røstad A (2013). Winter and spring diving behavior of bowhead whales relative to prey. Anim. Biotelemetry.

[36] Madsen SD, Nielsen TG, Hansen BW (2001). Annual population development and production by *Calanus finmarchicus*, *C. glacialis* and *C. hyperboreus* in Disko Bay, western Greenland. Mar. Biol..

[37] Hansen MO, Nielsen TG, Stedmon CA, Munk P (2012). Oceanographic regime shift during 1997 in Disko Bay, Western Greenland. Limnol. Oceanogr..

[38] Andersen, O. *The Annual Cycle of Temperature, Salinity, Currents and Water Masses in Disko Bugt and Adjacent Waters, West Greenland*. **5**, (Museum Tusculanum Press, 1981).

[39] Ribergaard, M. H., Olsen, M. & Mortensen, S. *Oceanographic investigations off West Greenland 2007*. (NAFO Scientific Council Documents, 2008).

[40] Sheldon CM (2015). Variable influx of West Greenland Current water into the Labrador Current through the last 7200 years: a multiproxy record from Trinity Bay (NE Newfoundland). arktos.

[41] Curry B, Lee CM, Petrie B, Moritz RE, Kwok R (2013). Multiyear Volume, Liquid Freshwater, and Sea Ice Transports through Davis Strait, 2004–10. J. Phys. Oceanogr..

[42] Reese CS, Calvin JA, George JC, Tarpley RJ (2001). Estimation of Fetal Growth and Gestation in Bowhead Whales. J. Am. Stat. Assoc..

[43] Finley KJ (2001). Natural History and Conservation of the Greenland Whale, or Bowhead, in the Northwest Atlantic. Arctic.

[44] Ferguson SH, Dueck L, Loseto LL, Luque SP (2010). Bowhead whale *Balaena mysticetus* seasonal selection of sea ice. Mar. Ecol. Prog. Ser..

[45] Nerini MK, Braham HW, Marquette WM, Rugh DJ (1984). Life history of the bowhead whale, Balaena mysticetus (Mammalia: Cetacea). J. Zool..

[46] George JC, Clark C, Carroll GM, Ellison WT (1989). Observations on the Ice-Breaking and Ice Navigation Behavior of Migrating Bowhead Whales (Balaena mysticetus) near Point Barrow, Alaska, Spring 1985. Arctic.

[47] Gosselin M, Levasseur M, Wheeler PA, Horner RA, Booth BC (1997). New measurements of phytoplankton and ice algal production in the Arctic Ocean. Deep Sea Res. Part II Top. Stud. Oceanogr..

[48] Heide-Jørgensen MP (2013). The Significance of the North Water Polynya to Arctic Top Predators. AMBIO.

[49] Munk P, Nielsen TG, Hansen BW (2015). Horizontal and vertical dynamics of zooplankton and larval fish communities during mid-summer in Disko Bay, West Greenland. J. Plankton Res..

[50] Heide-Jørgensen MP, Garde E, Nielsen N, Andersen O, Hansen S (2012). A note on biological data from the hunt of bowhead whales in West Greenland 2009–2011. J. Cetacean Res. Manag..

[51] Hokkanen JEI (1990). Temperature regulation of marine mammals. J. Theor. Biol..

[52] Mannocci L (2014). Predicting cetacean and seabird habitats across a productivity gradient in the South Pacific gyre. Prog. Oceanogr..

[53] Mannocci L (2014). Predicting top predator habitats in the Southwest Indian Ocean. Ecography.

[54] Lambert C, Mannocci L, Lehodey P, Ridoux V (2014). Predicting Cetacean Habitats from Their Energetic Needs and the Distribution of Their Prey in Two Contrasted Tropical Regions. PLOS ONE.

[55] Heide-Jørgensen MP, Laidre KL, Logsdon ML, Nielsen TG (2007). Springtime coupling between chlorophyll a, sea ice and sea surface temperature in Disko Bay, West Greenland. Prog. Oceanogr..

[56] Swalethorp R (2011). Grazing, egg production, and biochemical evidence of differences in the life strategies of Calanus finmarchicus, C. glacialis and C. hyperboreus in Disko Bay, western Greenland. Mar. Ecol. Prog. Ser..

[57] Dünweber M (2010). Succession and fate of the spring diatom bloom in Disko Bay, western Greenland. Mar. Ecol. Prog. Ser..

[58] Heide‐Jørgensen MP, Richard PR, Dietz R, Laidre KL (2012). A metapopulation model for Canadian and West Greenland narwhals. Anim. Conserv..

[59] NAMMCO. *Report of the Global Review of Monodontids* (2018).

[60] Richard PR, Martin AR, Orr JR (2001). Summer and Autumn Movements of Belugas of the Bastern Beaufort Sea Stock. Arctic.

[61] Dietz R (2008). Movements of narwhals (*Monodon monoceros*) from Admiralty Inlet monitored by satellite telemetry. Polar Biol..

[62] George JCCraig, Zeh J, Suydam R, Clark C (2004). Abundance and Population Trend (1978–2001) of Western Arctic Bowhead Whales Surveyed Near Barrow, Alaska. Mar. Mammal Sci..

[63] Nielsen NH, Laidre K, Larsen RS, Heide-Jørgensen MP (2015). Identification of Potential Foraging Areas for Bowhead Whales in Baffin Bay and Adjacent Waters. Arctic.

[64] Heide‐Jørgensen MP, Kleivane L, ØIen N, Laidre KL, Jensen MV (2001). A new technique for deploying satellite transmitters on baleen whales: tracking a blue whale (*Balaenoptera musculus* in the North Atlantic. Mar. Mammal Sci..

[65] R Core Team. R: A language and environment for statistical computing. R Foundation for Statistical Computing, Vienna, Austria. http://www.R-project.org/ (2017).

[66] Albertsen CM, Whoriskey K, Yurkowski D, Nielsen A, Flemming JM (2015). Fast fitting of non-Gaussian state-space models to animal movement data via Template Model Builder. Ecology.

[67] Chambault P (2017). The Gulf Stream frontal system: A key oceanographic feature in the habitat selection of the leatherback turtle?. Deep Sea Res. Part Oceanogr. Res. Pap..

[68] Eschricht, D. F. On the Greenland Right-Whale *(Balaena mysticetu*s). *Recent Mem. Cetacean* 1–145 (1866).

[69] Wood, S. *Generalized Additive Models: An Introduction with R*. (CRC Press, 2006).

[70] Wood SN, Pya N, Säfken B (2016). Smoothing Parameter and Model Selection for General Smooth Models. J. Am. Stat. Assoc..

[71] Lindén A, Mäntyniemi S (2011). Using the negative binomial distribution to model overdispersion in ecological count data. Ecology.

[72] Naimi B, Hamm NAS, Groen TA, Skidmore AK, Toxopeus AG (2014). Where is positional uncertainty a problem for species distribution modelling?. Ecography.

