# Supplementary material for: Sea surface temperature predicts the movements of an Arctic cetacean: the bowhead whale
Source: Sci Rep. 2018 Jun 25;8:9658. doi: 10.1038/s41598-018-27966-1 (PMC6018504; doi:10.1038/s41598-018-27966-1)
Supplement: Supplementary file 1 — Summary of the horizontal movements of the 98 bowhead whales (Table S1) and AIC results showing all possible combinations for the seasonal GAMs (Table S2). [file 41598_2018_27966_MOESM1_ESM.docx]

Sea surface temperature predicts the movements of an Arctic cetacean: the bowhead whale

Philippine Chambault^1*^, Christoffer Moesgaard Albertsen^2^, Toby A. Patterson^3^, Rikke G. Hansen^1^, Outi Tervo^1^, Kristin L. Laidre^4^, Mads Peter Heide-Jørgensen^1^

^1^ Greenland Institute of Natural Resources, Strandgade 91, 2, DK-1401, Copenhagen, Denmark.

^2^ National Institute of Aquatic Resources, Technical University of Denmark, DK-2800 Kongens Lyngby, Denmark.

^3^ CSIRO Oceans and Atmosphere, GPO Box 1538, Hobart, TAS 7000, Australia

^4^ Polar Science Center, Applied Physics Laboratory, University of Washington, Seattle, Washington, United States of America

* Corresponding author: philippine.chambault@gmail.com (PC)

Key words: seasonal pattern, satellite tracking, habitat affinities, SST front, sea ice edge, Baffin Bay, West Greenland current, hyperthermia

**Supplementary Information**

**Tabl**e **S1. Summary of the horizontal movements of the 98 bowhead whales tagged West of Greenland between 2011 and 2011.** Nloc refers to the number of locations recorded per whale**.**

| **Id** | **Start date** | **End date** | **Sex** | **Tag location** | **Nloc** | **Distance** | **Duration** | **Speed** |
| --- | --- | --- | --- | --- | --- | --- | --- | --- |
|  |  |  |  |  |  | **(km)** | **(d)** | **(km. ^-1^)** |
| 26712_01 | 06/05/2001 | 11/05/2001 | F | DB | 48 | 200 | 5 | 1.8±1.8 |
| 26716_01 | 07/05/2001 | 26/06/2001 | F | DB | 264 | 2,177 | 50 | 2.1±1.8 |
| 20158_02 | 08/05/2002 | 10/06/2002 | M | DB | 445 | 1464 | 33 | 1.6±0.9 |
| 20160_02 | 04/05/2002 | 17/11/2002 | M | DB | 501 | 5571 | 197 | 1±0.94 |
| 20685FB_02 | 05/07/2002 | 29/09/2002 | M | FB | 127 | 933 | 86 | 1.4±1.9 |
| 20688_02 | 08/05/2002 | 17/05/2002 | F | DB | 118 | 394 | 9 | 2.3±0.8 |
| 21794_02 | 10/05/2002 | 22/05/2002 | U | DB | 193 | 954 | 12 | 3±1.3 |
| 21803_02 | 12/05/2002 | 16/05/2002 | U | DB | 82 | 324 | 4 | 3.2±1.9 |
| 24641FB_02 | 05/07/2002 | 23/08/2002 | F | FB | 104 | 248 | 49 | 0.8±0.7 |
| 27262_02 | 12/05/2002 | 22/05/2002 | U | DB | 159 | 487 | 10 | 1.5±1 |
| 7933_02 | 07/05/2002 | 08/06/2002 | F | DB | 478 | 1,546 | 32 | 2±1.2 |
| 20160FB_03 | 04/07/2003 | 13/08/2003 | F | FB | 143 | 615 | 40 | 2±1.5 |
| 20167FB_03 | 07/07/2003 | 11/08/2003 | M | FB | 650 | 1,368 | 35 | 1.1±1.1 |
| 20688_03 | 11/05/2003 | 13/12/2003 | F | DB | 347 | 4,057 | 216 | 1.2±0.8 |
| 20696_03 | 31/05/2003 | 13/12/2003 | F | DB | 55 | 3,349 | 196 | 1.5±1.6 |
| 21802FB_03 | 11/07/2003 | 08/08/2003 | F | FB | 762 | 1,410 | 28 | 1.7±1.3 |
| 24641FB_03 | 08/07/2003 | 10/10/2003 | F | FB | 629 | 1,385 | 94 | 1.1±1 |
| 26712_03 | 17/05/2003 | 25/06/2003 | F | DB | 307 | 1,945 | 39 | 2.4±1.9 |
| 26712FB_03 | 18/07/2003 | 05/08/2003 | F | FB | 174 | 670 | 18 | 1.5±1.6 |
| 37229FB_03 | 13/07/2003 | 06/09/2003 | F | FB | 954 | 1,054 | 55 | 0.8±0.7 |
| 37230FB_03 | 04/07/2003 | 21/09/2003 | F | FB | 747 | 1,162 | 79 | 0.8±0.8 |
| 37231FB_03 | 04/07/2003 | 27/08/2003 | U | FB | 243 | 333 | 54 | 1.4±1.1 |
| 37280FB_03 | 04/07/2003 | 19/11/2003 | F | FB | 3,157 | 3,678 | 138 | 1.4±1.2 |
| 20157CS_04 | 27/05/2004 | 27/07/2004 | M | CS | 46 | 1,983 | 61 | 0.9±0.9 |
| 20167CS_05 | 15/07/2005 | 27/02/2006 | U | CS | 1,799 | 5,419 | 227 | 1.3±1.2 |
| 20687CS_05 | 10/07/2005 | 03/09/2005 | M | CS | 391 | 2,817 | 55 | 1.2±1.2 |
| 20690_05 | 29/04/2005 | 24/10/2005 | U | DB | 308 | 3,290 | 178 | 0.81±0.8 |
| 20693_05 | 23/04/2005 | 16/05/2005 | M | DB | 111 | 964 | 23 | 1.9±1.4 |
| 21791_05 | 26/04/2005 | 28/07/2005 | F | DB | 1,483 | 3,831 | 93 | 1.8±1.3 |
| 21792_05 | 26/04/2005 | 07/07/2005 | M | DB | 711 | 1,857 | 72 | 0.9±0.9 |
| 21794_05 | 27/04/2005 | 05/10/2005 | M | DB | 3,046 | 5,331 | 161 | 1.1±1.1 |
| 21800_05 | 27/04/2005 | 14/08/2005 | F | DB | 214 | 2,771 | 109 | 2.1±1.7 |
| 26715_05 | 22/04/2005 | 14/05/2005 | F | DB | 60 | 282 | 22 | 0.7±0.5 |
| 3965CS_05 | 19/07/2005 | 14/08/2005 | M | CS | 7 | 137 | 26 | 0.5±0.4 |
| 7929_05 | 16/04/2005 | 19/09/2005 | F | DB | 2,101 | 4,953 | 156 | 1.1±0.9 |
| 20158_06 | 02/05/2006 | 09/08/2006 | F | DB | 1,170 | 3,956 | 99 | 1.6±1.3 |
| 21791_06 | 16/04/2006 | 12/05/2006 | U | DB | 397 | 545 | 26 | 0.8±0.6 |
| 21794_06 | 16/04/2006 | 12/09/2006 | U | DB | 1,137 | 4,445 | 149 | 1.4±1.1 |
| 21800_06 | 17/04/2006 | 20/09/2006 | F | DB | 2,838 | 7,120 | 156 | 1.9±1.4 |
| 37231_06 | 23/04/2006 | 02/05/2006 | F | DB | 25 | 143 | 9 | 1.5±2.1 |
| 20164_08 | 23/04/2008 | 07/11/2008 | F | DB | 1,185 | 4,983 | 198 | 1.8±1.7 |
| 20165_08 | 26/04/2008 | 08/06/2008 | F | DB | 439 | 1,170 | 43 | 1.8±1.5 |
| 20166_08 | 29/04/2008 | 15/11/2008 | F | DB | 1,598 | 5,381 | 200 | 1.6±1.3 |
| 20168_08 | 28/05/2008 | 06/09/2008 | U | DB | 1,583 | 4,355 | 101 | 1.8±1.3 |
| 20169_08 | 28/05/2008 | 10/08/2008 | U | DB | 647 | 3,308 | 74 | 2.1±1.7 |
| 20683_08 | 28/05/2008 | 06/10/2008 | U | DB | 771 | 4,552 | 131 | 1.6±1.5 |
| 20690_08 | 03/06/2008 | 26/09/2008 | U | DB | 1,609 | 3,236 | 115 | 1.2±1.1 |
| 6337_08 | 20/04/2008 | 31/05/2008 | M | DB | 36 | 539 | 41 | 0.9±1.1 |
| 7930_08 | 25/04/2008 | 29/05/2008 | U | DB | 565 | 1,543 | 34 | 2±1.5 |
| 7934_08 | 21/04/2008 | 27/06/2008 | F | DB | 145 | 1,815 | 67 | 1.7±1.6 |
| 20157_09 | 27/05/2009 | 03/08/2009 | M | DB | 108 | 2,295 | 68 | 1.5±1.4 |
| 20162_09 | 29/05/2009 | 11/05/2010 | U | DB | 1,108 | 9,595 | 347 | 1.5±1.3 |
| 20167_09 | 17/05/2009 | 14/07/2010 | F | DB | 1,678 | 7,604 | 423 | 1.1±1.1 |
| 20685_09 | 13/06/2009 | 27/01/2010 | F | DB | 1,956 | 8,960 | 228 | 1.8±1.6 |
| 20688_09 | 14/05/2009 | 11/06/2009 | F | DB | 105 | 1,475 | 28 | 2.6±1.6 |
| 20689_09 | 13/05/2009 | 29/11/2009 | U | DB | 1,458 | 4,631 | 200 | 1.7±1.2 |
| 20696_09 | 09/04/2009 | 16/08/2009 | F | DB | 1,082 | 3,689 | 129 | 1.9±1.5 |
| 21791_09 | 01/05/2009 | 01/09/2009 | F | DB | 827 | 3,179 | 123 | 1.3±1.1 |
| 21794_09 | 15/05/2009 | 30/07/2009 | F | DB | 574 | 2,886 | 76 | 1.5±1.2 |
| 21802_09 | 18/05/2009 | 16/12/2009 | F | DB | 1,818 | 5,131 | 212 | 1.4±1.2 |
| 21803_09 | 16/05/2009 | 03/07/2009 | F | DB | 718 | 2,297 | 48 | 1.9±1.3 |
| 24642_09 | 02/05/2009 | 12/06/2009 | U | DB | 385 | 995 | 41 | 0.8±1.1 |
| 26715_09 | 09/07/2009 | 07/09/2009 | F | DB | 556 | 2,593 | 60 | 1.6±1.3 |
| 27258_09 | 05/06/2009 | 16/06/2009 | F | DB | 121 | 859 | 11 | 3.7±2.3 |
| 7925_09 | 22/05/2009 | 24/06/2009 | F | DB | 14 | 1,009 | 33 | 0.7±0.4 |
| 7927_09 | 25/05/2009 | 30/12/2009 | F | DB | 1,252 | 7,788 | 219 | 1.7±1.4 |
| 7929_09 | 13/05/2009 | 12/06/2009 | F | DB | 319 | 1,084 | 30 | 1.9±1.6 |
| 7930_09 | 26/05/2009 | 24/06/2009 | M | DB | 152 | 1,205 | 29 | 1.8±1.3 |
| 20164_10 | 02/04/2010 | 23/01/2011 | F | DB | 100 | 3,879 | 296 | 2.1±1.5 |
| 20165_10 | 02/04/2010 | 26/05/2010 | F | DB | 885 | 707 | 54 | 0.8±0.5 |
| 20166_10 | 02/04/2010 | 12/08/2010 | F | DB | 1,378 | 3,437 | 132 | 1.7±1.4 |
| 20168_10 | 02/04/2010 | 11/06/2010 | F | DB | 348 | 1,133 | 70 | 1.4±1.1 |
| 20685_10 | 08/04/2010 | 24/08/2010 | F | DB | 1,124 | 3,887 | 138 | 1.6±1.2 |
| 20690_10 | 20/04/2010 | 21/06/2010 | F | DB | 833 | 1,768 | 62 | 1.5±1.1 |
| 21793_10 | 20/05/2010 | 13/07/2010 | U | DB | 491 | 1,969 | 54 | 1.6±1.3 |
| 21803_10 | 27/04/2010 | 09/08/2010 | U | DB | 663 | 2,775 | 104 | 1.6±1.3 |
| 21810_10 | 15/04/2010 | 12/10/2010 | M | DB | 853 | 3,272 | 180 | 1.4±1.3 |
| 22850_10 | 21/04/2010 | 01/08/2010 | F | DB | 806 | 2,794 | 102 | 1.7±1.4 |
| 22854_10 | 07/05/2010 | 14/08/2010 | F | DB | 1,325 | 4,041 | 99 | 1.6±1.2 |
| 24638_10 | 02/05/2010 | 14/06/2010 | F | DB | 169 | 920 | 43 | 1.3±1.1 |
| 24640_10 | 29/04/2010 | 31/05/2010 | F | DB | 98 | 495 | 32 | 2.0±1.7 |
| 27258_10 | 31/03/2010 | 02/09/2010 | F | DB | 1,640 | 5,532 | 155 | 1.7±1.4 |
| 27262_10 | 19/03/2010 | 21/07/2011 | F | DB | 1,508 | 8,835 | 489 | 1.2±0.9 |
| 37227_10 | 17/03/2010 | 14/05/2011 | F | DB | 4,129 | 15,230 | 423 | 1.9±1.4 |
| 37228_10 | 31/03/2010 | 28/10/2010 | F | DB | 1,890 | 7,187 | 211 | 1.8±1.4 |
| 37235_10 | 15/04/2010 | 23/03/2011 | M | DB | 3,264 | 13,826 | 342 | 2.3±1.6 |
| 37282_10 | 20/03/2010 | 20/08/2010 | F | DB | 1,392 | 3,843 | 153 | 1.4±1.2 |
| 42524_10 | 31/03/2010 | 08/08/2010 | F | DB | 723 | 3,632 | 130 | 1.7±1.3 |
| 50687_10 | 02/04/2010 | 27/09/2010 | F | DB | 642 | 3,585 | 178 | 1.4±1.2 |
| 60005_10 | 04/04/2010 | 19/07/2010 | F | DB | 997 | 3,156 | 106 | 1.4±1.3 |
| 6337_10 | 07/04/2010 | 13/06/2010 | U | DB | 493 | 1,652 | 67 | 1.7±1.6 |
| 7618_10 | 04/04/2010 | 09/06/2010 | F | DB | 584 | 1 535 | 66 | 0.9±0.8 |
| 7927_10 | 19/04/2010 | 09/12/2010 | M | DB | 2,353 | 6,862 | 234 | 1.5±1.3 |
| 7929_10 | 03/05/2010 | 07/11/2010 | M | DB | 1,783 | 9,097 | 188 | 2.4±1.8 |
| 7930_10 | 09/05/2010 | 01/09/2010 | F | DB | 514 | 3,554 | 115 | 1.6±1.2 |
| 93116_10 | 23/04/2010 | 12/08/2010 | M | DB | 600 | 3,610 | 111 | 1.9±1.4 |
| 6335_11 | 08/05/2011 | 08/05/2011 | F | DB | 17 | 32 | 1 | 2.9±1.9 |
| 7617_11 | 08/05/2011 | 08/05/2011 | M | DB | 2 | 5 | 1 | 1.4±2.0 |
|  |  |  |  |  | 826±821 | 3118±2815 | 110±97 | 1.5±0.3 |

**Table S2**. **AIC results showing all possible combinations for the seasonal GAMs relating whale’s density to Sea Surface Temperature (SST), Sea Ice Concentration (SIC), distance to SST front (distToFront) and distance to sea ice edge (distToSeaIce).** The presented combinations were previously tested for collinearity and the rows in bold refer to the best model for each season.

| **Model** | **AIC** | **Δ AIC** | **Explained deviance (%)** |
| --- | --- | --- | --- |
| ***SPRING*** |  |  |  |
| **SST+distToFront+distToSeaIce** | **14247** | **0** | **50.4** |
| SIC+distToFront+distToSeaIce | 14252 | 5 | 50.0 |
| distToFront+distToSeaIce | 14259 | 12 | 50.1 |
| SST+distToSeaIce | 14260 | 13 | 50.1 |
| SIC+distToSeaIce | 14260 | 13 | 49.8 |
| SST+SIC+distToFront | 14270 | 23 | 49.2 |
| SST+SIC | 14272 | 25 | 49.3 |
| distToSeaIce | 14280 | 33 | 49.6 |
| SST+distToFront | 14290 | 43 | 49.0 |
| SIC+distToFront | 14290 | 43 | 48.3 |
| SIC | 14324 | 77 | 48.2 |
| distToFront | 14333 | 86 | 47.6 |
| SST | 14348 | 101 | 48.0 |
| ***SUMMER*** |  |  |  |
| **SST+distToSeaIce** | **8033** | **0** | **45.3** |
| SST+distToSeaIce+distToFront | 8036 | 3 | 45.3 |
| SIC+distToSeaIce | 8036 | 3 | 45.0 |
| SST | 8037 | 4 | 45.0 |
| SIC | 8038 | 5 | 45.0 |
| distToSeaIce+distToFront | 8038 | 5 | 45.1 |
| distToSeaIce | 8039 | 6 | 44.9 |
| SIC+SST | 8039 | 6 | 45.0 |
| SST+distToFront | 8039 | 6 | 45.0 |
| distToFront | 8039 | 6 | 44.9 |
| SIC+distToFront | 8040 | 7 | 44.9 |
| distToFront+distToSeaIce | 8041 | 8 | 44.9 |
| SST+SIC+distToFront | 8041 | 8 | 45.0 |
| ***AUTUMN*** |  |  |  |
| **SST** | **3038** | **0** | **31.5** |
| SST+distToFront | 3085 | 47 | 32.5 |
| SST+SIC | 3086 | 48 | 31.5 |
| SST+distToFront+distToSeaIce | 3086 | 48 | 32.4 |
| distToFront | 3088 | 50 | 31.3 |
| SST+SIC+distToFront | 3086 | 48 | 32.5 |
| SST+distToSeaIce | 3090 | 52 | 29.5 |
| distToFront+distToSeaIce | 3091 | 53 | 31.2 |
| distToFront+SIC | 3093 | 55 | 29.3 |
| distToSeaIce+SIC+distToFront | 3093 | 55 | 29.4 |
| SST+SIC+distToSeaIce | 3094 | 56 | 29.3 |
| distToSeaIce+SIC | 3095 | 57 | 28.2 |
| SIC | 3096 | 58 | 27.5 |
| distToSeaIce | 3100 | 62 | 29.3 |
| ***WINTER*** |  |  |  |
| **SST+distToFront+distToSeaIce** | **1263** | **0** | **58.0** |
| SST+distToSeaIce | 1270 | 7 | 54.3 |
| distFront+distToSeaIce | 1274 | 11 | 47.4 |
| distToSeaIce | 1276 | 13 | 49.8 |
| distToFront+SIC+SST | 1279 | 16 | 44.7 |
| SST+distToFront | 1280 | 17 | 43.5 |
| distToSeaIce+SIC | 1280 | 17 | 49.5 |
| SST+SIC | 1282 | 19 | 48.5 |
| SST | 1284 | 21 | 46.7 |
| distToSeaIce+SIC+distToFront | 1287 | 24 | 41.1 |
| SIC | 1288 | 25 | 44.0 |
| distToFront | 1288 | 25 | 42.4 |
| distToFront+SIC | 1292 | 29 | 38.6 |
